# The development of Cognitive Behavioural Therapy (CBT) for chronic loneliness in children and young people: Protocol for a single-case experimental design

**DOI:** 10.1371/journal.pone.0278746

**Published:** 2022-12-09

**Authors:** Tom Cawthorne, Anton Käll, Sophie Bennett, Gerhard Andersson, Roz Shafran

**Affiliations:** 1 Royal Holloway, University of London, Egham, United Kingdom; 2 Camden and Islington NHS Foundation Trust, London, United Kingdom; 3 Department of Behavioural Sciences and Learning, Department of Biomedical and Clinical Sciences, Linköping University, Linköping, Sweden; 4 UCL Great Ormond Street Institute of Child Health, London, England; 5 Department of Clinical Neuroscience, Karolinska Institute, Stockholm, Sweden; National Institute of Mental Health, UNITED STATES

## Abstract

Loneliness is a significant problem for young people and is associated with a range of physical and mental health difficulties. Meta-analyses have identified that interventions aimed at young people who report loneliness as their primary problem are lacking within the literature. In adults, the most effective interventions for loneliness are those which target the underlying maladaptive social cognitions. Therefore, we have developed a modular Cognitive Behavioural Therapy (CBT) intervention for children and young people. The aim of this study is to conduct a multiple baseline single-case experimental design (SCED) to assess the efficacy, feasibility and acceptability of this intervention. In total 6–8 11–18-year-olds and their families will be recruited. The design consists of AB+ post-intervention, where A is the baseline phase, B is the intervention phase and then a post-intervention phase. Participants will complete a baseline assessment, before being randomised to one of four different baseline lengths (12 days, 19 days, 26 days or 33 days). Participants will then complete an average of 12 sessions of CBT, with the aim being to reduce their feelings of loneliness. Participants will then complete a 12-day post-intervention phase. Participant loneliness will be repeatedly assessed throughout the three phases of the intervention using the Three-item Loneliness Scale, which will be the primary outcome. Secondary outcomes will be reliable and clinically meaningful change on the UCLA Loneliness Scale, Revised Child Anxiety and Depression Scale (RCADS) and Strengths and Difficulties Questionnaire (SDQ). Feasibility and participant satisfaction will also be assessed and reported.

**Trial registration: ClinicalTrails.gov trial registration number:** NCT05149963 (Date registered: 07.12.2021). https://www.clinicaltrials.gov/ct2/show/NCT05149963?term=cbt&cond=loneliness&draw=2&rank=1.

## Introduction

Loneliness is defined as the discrepancy between actual and desired social interaction [1]. Temporary loneliness is a normal phenomenon and regarded as part of everyday life [1, 2]. Research suggests that spending time alone is a normative adolescent experience and part of the developmental task of becoming independent, with some young people reporting time alone to be important for the development of self-identity [3]. This is differentiated from chronic loneliness, where an individual experiences a lack of satisfying relationships over time [4].

Chronic loneliness is a transdiagnostic problem and associated with a range of physical and mental health difficulties [5, 6]. Loneliness also increases the likelihood of mortality by 26% even when controlling for multiple covariates [7]. Loneliness in children and young people is associated with higher levels of peer victimisation [8, 9], lower self-worth and a range of mental and physical health problems [10–14]. The population of young people who are at an elevated risk of loneliness is highly heterogenous. It includes those with chronic health problems [15], mental health difficulties [10, 11] and those on the autism spectrum [16]. It is also hypothesised that interventions aimed at reducing loneliness may be an important active ingredient in the prevention and treatment of anxiety and depression in young people [17].

Distinct developmental trajectories of youth loneliness have been identified. It has been found that 18% of young people present with moderate levels of loneliness which increase over time and 22% present with consistently high levels of loneliness throughout childhood [14]. These findings are of significant concern, as it suggests that for many young people loneliness does not spontaneously remiss without intervention. Loneliness in young people is a significant challenge cross-culturally [18], including within the United Kingdom where 10% of children are reported to often experience loneliness, with 14% of those aged 10–12 and 8.6% of those aged 13–15 reporting that they “often” feel lonely [19].

Psychological interventions can be effective in reducing loneliness across the lifespan [20]. A recent meta-analysis of interventions for loneliness in young people highlighted a range of approaches that may reduce loneliness as a secondary outcome in at-risk groups [21]. These included intrapersonal strategies (e.g., psychological therapy), interpersonal strategies (e.g., social skills interventions), social strategies (e.g., social support) and self-help strategies (e.g., therapeutic apps). However, interventions specifically aimed at young people who report loneliness as their primary difficulty (rather than those at-risk of loneliness) are lacking within the literature. The authors also highlighted that current interventions do not differentiate between transient and chronic loneliness; with the latter requiring interventions targeting the underlying anxieties and negative cognitive biases that maintain chronic loneliness. A further limitation of the current evidence base is the lack of controlled experimental designs, which limits the conclusions that can be drawn regarding the efficacy of reported interventions.

A meta-analytic review of adult loneliness interventions by Masi et al. [22] identified that the most efficacious interventions were those which targeted the underlying maladaptive social cognitions. This may also be the case for youth populations and indicates that interventions aimed specifically at reducing loneliness in children and young people should be based within a Cognitive Behavioural Therapy (CBT) framework. However, this finding was not replicated in a recent systematic review and meta-analysis by Hickin et al. [20], which found CBT to be the third most effective treatment approach behind a reminiscence intervention for older adults and social identity interventions. This suggests that interventions for loneliness should take a modular approach, incorporating different evidence-based treatment components which can be selected based upon treatment need. A modular approach may be particularly appropriate for the adolescent population, due to the high levels of heterogeneity in the presentations of young people presenting with chronic loneliness [10, 11, 15, 16].

Käll et al. [23] have developed a modular cognitive behavioural analysis of chronic loneliness based upon a common elements approach. They identified the common practice elements within existing interventions shown to reduce loneliness and identified the ‘target’ mechanisms for a modular loneliness intervention. The modular model has varying treatment implications and its modular nature means it can be applied flexibly to different populations. A modular approach may be particularly appropriate for this client group, as the population is highly heterogenous and it is not yet known what interventions work for whom [17]. Interventions informed by this modular formulation have since been shown to be efficacious in two online randomised controlled trials (RCT) for reducing loneliness in adulthood [24, 25]. This suggests that this formulation model provides a good underlying theoretical basis for the development of Cognitive Behavioural Therapy (CBT) for loneliness in children and young people.

Associations between parental and child mental health problems have been identified, with parental mental health problems predictive of poorer CBT outcomes in young people [26, 27]. Therefore, in this present study parental health will also examined, to assess whether there is preliminary evidence for it having a role in treatment response.

The modular CBT intervention developed for this study will be evaluated through a multiple baseline single-case experimental design (SCED) [28–30]. A criticism of previous loneliness interventions for children and young people has been the lack of controlled experimental designs [21]. SCED’s provide a controlled experimental approach from which causal inferences can be drawn and give the detail and richness commonly associated with case studies [28]. As this is the first study to test this intervention, an iterative approach will be taken to development based upon Plan-Do-Study-Act (PDSA) cycles [31]. PDSA cycles are an individualised quality improvement method which aims to maximise the feasibility and acceptability of interventions by providing a framework for development, testing and implementation of change [32]. This approach has also been used effectively in previous intervention development studies for children and young people [33].

### Aims and objectives

The primary objective will be to evaluate the efficacy of CBT for loneliness in children and young people ages 11–18. The primary outcome will be differences in scores on the Three-Item Loneliness Scale [34] between baseline and intervention and baseline and post-intervention. It is hypothesised that there will be a significant decrease in self-reported loneliness across both time-points. The secondary outcomes will be self-reported loneliness scores on the UCLA Loneliness Scale (UCLA-LS-3) [35] and self and parent-reported scores on the Strengths and Difficulties Questionnaires (SDQ) [36] and Revised Child Anxiety and Depression Scale (RCADS) [37]. It is hypothesised that there will be a reliable and clinically meaningful change [38] in total loneliness scores on the UCLA-LS-3, in the impact scores on the SDQ and in total anxiety and depression scores on the RCADS.

The secondary objective is to evaluate the feasibly and acceptability of the intervention process. This will be defined in terms of a) meeting the recruitment target, b) participant retention and c) participant feedback of the intervention using the Experience of Service Questionnaire [39].

## Methods

### Study design

The efficacy of CBT for loneliness in children and young people will be evaluated through a randomised multiple-baseline single-case experimental design (SCED) [28–30]. The design consists of AB+ post-intervention, where A is the baseline phase, B is the intervention phase and then a post-intervention phase. In the SCED approach participants are repeatedly assessed on at least one independent variable, in this case self-reported loneliness on the Three-item Loneliness Scale [34], across each phase of the intervention. This repeated measurement and within subject replication is then used to test the effects of the intervention for individual participants [28, 30]. Multiple-baseline SCEDs are a controlled experimental approach, with multiple-baselines protecting against threats to internal validity [40].

The construction of this SCED trial and the reporting of the results will be in accordance with the Single-Case Reporting Guidelines in Behavioural Intervention (SCRIBE) [30].

The study has received ethical approval from Royal Holloway, University of London on 22.03.2021 (ethical approval number: 2489). The study was registered with ClinicalTrials.gov on 07.12.2021 (registration number: NCT05149963).

### Procedure

**Fig 1:**
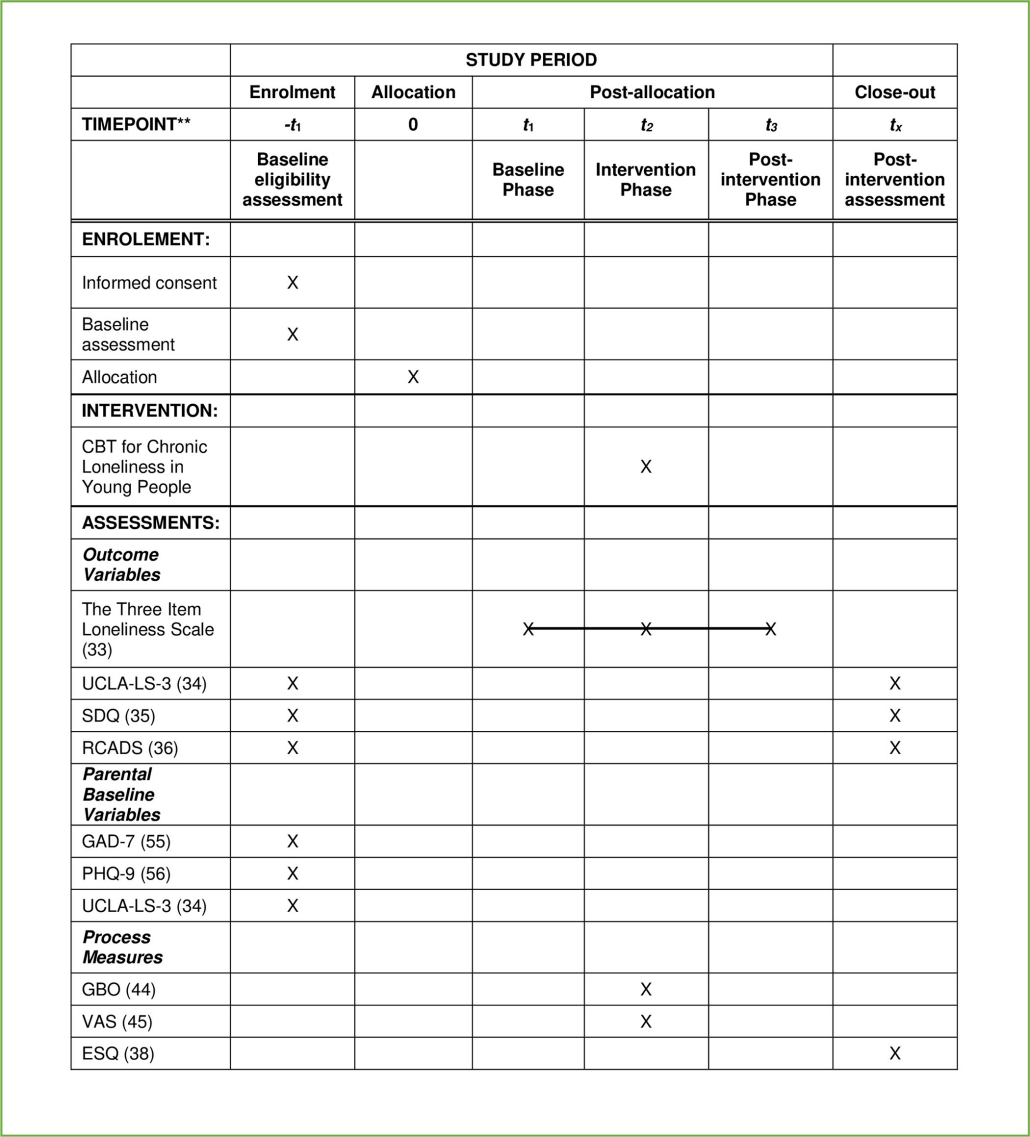
Showing the trials schedule of enrolment, interventions and assessments.

The trial procedure is shown in the schedule of enrolment, interventions and assessments (Fig 1).

Participants will be recruited via advertisements emailed to schools, shared via social media and word of mouth. Participants and their parents/carers will be sent an electronic information sheet and given the opportunity to ask any questions before completing an online consent form. Participants will provide informed ascent as they are under 18, with parents/carers providing informed consent. Participants and their parents/carers will then complete a baseline eligibility assessment. They will complete a range of online questionnaires via Qualtrics [41], a secure online platform. They will then attend a remote research assessment appointment to determine whether the family meet the eligibility criteria. The researcher completing the baseline and post-intervention assessments will not be involved in the clinical intervention and will be blinded to baseline allocation. After all baseline assessments for the participant group are completed, they will be discussed within supervision to confirm that the participants meet eligibility criteria. Eligible participants will then be randomised using a random number generator to one of four different baseline lengths (12 days, 19 days, 26 days or 33 days [42]. The decision to have a minimum of 12 observations per phase meets the requirements for guidelines and standards [43] and will allow for sufficient power for Tau-U analysis [44, 45].

Each of the families will then be contacted to inform them of their allocation. Randomisation and contacting of the families will be completed by a third researcher, who is not involved in either the clinical intervention or research assessments. Families will receive a £25 voucher for completing the baseline eligibility assessment. A letter will also be sent to their General Practitioner (GP) informing them of the family’s participation in the research study. The setting for the entire study will be remote via Zoom [46], with participants recruited from across the United Kingdom (UK).

All participants in the group will begin their baseline phase concurrently. During the baseline phase participants will be asked to complete the Three-Item Loneliness Scale [34] each day via Qualtrics [41]. SMS message reminders will also be sent to the young people and their parent/carers each day.

The baseline phase will be immediately followed by the intervention. Each family will receive (on average) 12 sessions of CBT for loneliness in children and young people. Where possible the first 4 sessions will take place bi-weekly, the second 4 sessions weekly and the final 4 sessions fortnightly.

For all participants the first two sessions will be focussed on assessment and then formulation. The final session for all participants will focus on relapse prevention. During the assessment session the participant will be asked to define 3 goal-based outcomes (GBOs) [47] for how they hope the intervention will reduce their loneliness, which will then guide the treatment process. During the intervention phase the participants will complete routine outcome measures (ROMs) for each session. This will include the Three-Item Loneliness Scale [34], visual analogue scales (VAS) of mood, anxiety and loneliness and ratings of their goal-based outcomes [48]. The ROMs will be completed via Qualtrics [41] before each session. If participants have not completed the ROMs before their appointment, then they will be completed with the clinician at the start of the session.

Participants will begin the post-intervention phase immediately after the final session of their intervention. During the post-intervention phase participants will be asked to complete the primary outcome measure, Three-Item Loneliness Scale [34], each day for 12 days. They will then complete their post-intervention assessment with the second researcher. Before the assessment they will be asked to re-complete the baseline questionnaires in addition to the parent and child-report versions of the Experience of Service Questionnaire [39] and a questionnaire asking whether COVID-19 or any other events have affected them during the intervention period. The family will be given a £25 voucher for completing the post-intervention assessment. A letter will be sent to their General Practitioner (GP) informing them of their participation in the research study has finished.

### Procedural fidelity

The intervention will be conducted by the first author (TC). He will receive weekly supervision from RS, AK and SB throughout the research and intervention process to ensure fidelity to the agreed protocol. Procedural fidelity during the intervention phase will also be monitored through the completion of adherence to the manual checklists after each intervention appointment.

### Participants

We will aim to recruit 6–8 participants to the study. All participants will be recruited from within the United Kingdom. Participants must meet the following inclusion/exclusion criteria:

#### Inclusion criteria

1. Aged 11–18
2. Score more than 42 on the UCLA-LS-3 [35], which is more than one standard deviation above the mean in a large community adolescent sample [49].
3. Have a parent/carer who is willing to take part in the study.
4. Report loneliness as their primary difficulty (i.e., they are able to identify relevant goal-based outcomes and their current difficulties are not attributable to a significant mental health problem).

#### Exclusion criteria

1. Currently receiving psychological therapy.
2. Started taking antidepressant in the last 8 weeks.
3. Eligibility assessment indicates a severe mental health problem not considered suitable for the trial intervention due to the clinical need for immediate intervention, e.g., active suicidality or psychosis.
4. Refusal for therapy sessions to be video recorded.
5. Having an intellectual disability at a level whereby they cannot access the intervention.
6. Do not have access to a laptop or smartphone which they can use for video calls.

### Intervention

To the best of our knowledge this is the first study to examine the efficacy of cognitive behavioural therapy for loneliness in children and young people. The intervention has been developed for this research study using a modular approach based upon Käll et al.’s [23] modular cognitive behavioural formulation. It incorporates translated elements of Käll et al., [25] online intervention, and is informed by the Modular approach to therapy for children with anxiety, depression, trauma, or conduct problems (MATCH-ADTC) [50], Groups4Health [51], PEERS social skills training [52], CBT for Social Anxiety Disorder for adolescence [53] and the literature implicating social camouflaging in mental health difficulties for those on the autism spectrum [54].

The intervention is being developed iteratively based upon feedback and clinical experience using PDSA cycles [31]. The current manual is comprised of 10 treatment modules (Table 1). All participants will complete Module 1 (Assessment), Module 2 (Formulation and Psychoeducation) and Module 10 (Relapse prevention). Other intervention modules will be chosen in collaboration with the participants based upon their personalised formulation and treatment goals. The number of sessions delivered for each module will be determined by treatment priorities, individual progress and the number of sessions remaining. The intervention will be delivered by the first author (TC) who is a trainee clinical psychologist. He will receive weekly supervision from the other authors (RS, AK, SB) who are all qualified clinical psychologists. If there is deterioration in wellbeing or risk issues are identified local statutory or healthcare services will be contacted as appropriate. Participants are able to withdraw from the trial at any time.

**Table 1:** Showing the treatment modules which comprise CBT for chronic loneliness in children and young people.

Module	Description
1. Assessment	The aim of this module is to complete a structured CBT assessment of the participants loneliness. The CBT assessment will cover each of the modules within Käll et al., [23] formulation. The assessment will also include questions regarding the participants development/early experiences, current living situation, education, physical health, lifestyle (diet, sleep, exercise, hobbies/interests, social media use) and a risk assessment, before identifying three goals for treatment.
2. Formulation and psychoeducation	The aim of this module is to collaboratively complete a modular formulation of the factors maintaining the participants chronic loneliness [23]. The participant will also be given psychoeducation on loneliness, the intervention process and will collaboratively develop a treatment plan based on their formulation and goal-based outcomes.
3. Challenging negative interpersonal appraisals and counterproductive behaviours	The aim of the module is to change the negative thought patterns and counter-productive behaviours which are maintaining chronic loneliness. Participants will be given psychoeducation on the CBT model and the relationship between thoughts, behaviours, physical feelings, and emotions. Participants will be supported to identify the negative interpersonal appraisals and counterproductive behaviours which are maintaining their loneliness. The participant will then be supported to develop within-session behavioural experiments to challenge the counterproductive processes that are maintain their loneliness, which will then be reinforced through agreed homework tasks.
4. Challenging negative thoughts and cognitive biases	The aim of the module is to challenge the negative cognitions and cognitive biases that are maintaining chronic loneliness. Participants will be given psychoeducation on automatic thought processes, negative thinking traps and their role in chronic loneliness. The participant will be supported to develop strategies to challenge thinking errors. The module is based upon the Cognitive Coping modules of MATCH-ADTC [50].
5. Challenging self-focussed attention, hypervigilance and camouflaging	The aim of the module is to challenge self-focussed attention, hypervigilance and/or camouflaging when they are maintaining chronic loneliness. The module is comprised of three submodules: 5A, Reducing Self-Focussed Attention, 5B Reducing Hypervigilance and 5C Reducing Camouflaging Behaviours. Each submodule provides psychoeducation on the role of that difficulty in maintaining chronic loneliness. The participant will then be supported to develop within-session behavioural experiments to challenge the counterproductive processes, which will then be reinforced through agreed homework tasks. Module 5A is based upon CBT for Social Anxiety in Adolescence [53], with module 5B being a hypervigilance-focussed adaptation of this. Module 5C is a novel module informed by the literature regarding the role of camouflaging in mental health difficulties in those on the autism spectrum [54].
6. Values-based social skills training	The aim of this module is to identify the participants social communication strengths and areas for development. The participant will then be supported to develop their social skills in line with their values and treatment goals aiming to reduce their loneliness. The module is informed by the UCLA PEERS program [52] and the social skills module of the internet-based CBT for loneliness in adults [24]. The module is comprised of four submodules: 6A Conversation skills, 6B Managing Teasing and Bullying, 6C Managing disagreements and 6D Dating and Flirting. An emphasis on remote communication is embedded across all submodules.
7. Problems solving	The aim of the module is to teach formal problem-solving to overcome practical barriers to reducing loneliness. The module is based upon the Problem-Solving module within MATCH-ADTC [50]. Participants will be provided with psychoeducation on the problem-solving STEPS before having the opportunity to practice the skills within the session. The participant will then be supported to use the problem-solving skills to overcome the practical barriers that are maintaining their loneliness.
8. Finding Friends	The aim of the module is to map the young person’s current social world and identify opportunities for the development of positive social relationships. Participants will be supported to map their current relationships and rate them in terms of positivity, similarity, support and time spent together. Participants will then link their current social groups based on compatibility. Participants will then be supported to identify strategies to improve their connection with current groups, as well as develop connections with new social groups. The module is informed by the Groups4Health intervention [51].
9. Managing Emotions	The aim of the module is to develop strategies to manage emotional responses where they are maintaining chronic loneliness. The module is comprised of two submodules; 9A: Activity selection (for low mood) and 9B Learning to relax (for anxiety management). Both submodules are based on MATCH-ADTC [50].
10. Relapse Prevention	The aim of the module is to develop a relapse prevention plan with the young person and their parent/carer. This will focus on what the family has learnt during the sessions, strategies to maintain their progress and overcome future difficulties and the difference between a lapse and relapse. Participants will be supported to complete a written plan in addition to a relapse prevention video.

### Outcome measures

#### Loneliness

*The three item loneliness scale [34].* This will be used to assess the child/young persons’ self-reported loneliness throughout each of the three phases of the study. The scale is a brief 3-item measure derived from the UCLA-LS-3 [35]. The measure contains three items rated on a 5-point Likert scale (0 = never, 1 = rarely, 2 = sometimes, 3 = often, 4 = very often). Answers are summed to a total score of 0–12, with higher scores indicating a higher level of loneliness. The Office for National Statistics (ONS) have validated a 3-reponse version of this measure with young people ages 10–15 [19]. In qualitative testing of the measure, they identified that the words “companionship” and “isolation” were difficult for some young people to understand. These changes in wording were also used in this study as the age range was similar to that used in the ONS validation. The items used were “How often do you feel that you have no one to talk to?”, “How often do you feel left out?” and “How often do you feel alone?”.

*UCLA Loneliness Scale (UCLA-LS-3) [35].* The measure will be used to assess the child/young person’s subjective experience of loneliness. The instrument consists of 20 items measured on a 4-point scale where the respondents are asked to indicate how frequently the statement is descriptive of them with the options of never, rarely, sometimes, and often. The UCLA-LS-3 has been used extensively in loneliness research, including in intervention studies [25, 55] and has been validated in a large adolescent sample [49]. The psychometric properties include a very high internal consistency (Cronbach’s α’s ranging from .89 to .94) and a good test-retest-reliability (.73 over a 1-year period) [35].

#### Psychological wellbeing

*The Strengths and Difficulties Questionnaires (SDQ) [36].* The self-report and parent-report versions will be used to assess the child’s psychological wellbeing. It is a brief behavioural screening questionnaire for children and young people ages 2 -17. The 25 items are divided between 5 scales (emotional, conduct problems, hyperactivity/inattention, peer relationship problems, prosocial behaviour), with the first four summed to provide a total difficulties score. The SDQ has an ‘impact scale’, which assesses the impact that symptoms have on everyday life in a range of domains (home, school, leisure). The SDQ has good internal consistency (mean Cronbach α .73), cross-informant correlation (mean 0.34), and retest stability after 4 to 6 months [36].

*The Revised Child Anxiety and Depression Scale (RCADS)*. The parent and self-report versions will be used to assess the child’s anxiety and depression. It is a 47-item questionnaire for 8–18-year-olds. Its subscales include separation anxiety disorder, social phobia, generalized anxiety disorder, panic disorder, obsessive compulsive disorder, and low mood (major depressive disorder). In addition to the subscales, a total anxiety scale and total anxiety and depression scale can be calculated. It has good reliability on subscales and total scale, with internal consistency of adequate-excellent across the different subscales [56]. It also had good test-retest reliability and good convergent and concurrent validity [56, 57].

#### Sample characterisation

*Demographic questionnaire*. Parents/carers will be asked to complete a demographic questionnaire. This will ask about the child’s age, gender, ethnic background, existing child psychiatric diagnoses, parent/carer’s age, family composition, parent/carer’s education level, parent/carer employment status and household income band.

The Generalised Anxiety Disorder Assessment (GAD-7) [58]

The measure will be used to assess the parents/carers self-reported anxiety. The GAD-7 is a 7-item measure with scores ranging from 0–21. Scores of 5, 10, and 15 represent cut-points for mild, moderate, and severe anxiety, respectively. The measure has been shown to have excellent internal consistency and good test-retest reliability and convergent validity [58].

The Patient Health Questionnaire (PHQ-9) [59]

The measure will be used to assess the parent/carers self-reported depression. It is a 9-item measure of depressive symptoms with scores ranging from 0–27. Each item asks the individual to rate the severity of their symptoms over the past two weeks. Scores of 5, 10, and 15 and 20 represent cut-points for mild, moderate, moderately severe and severe depression respectively. The PHQ-9 has demonstrated good convergent and discriminant validity and high internal consistency [59–61].

*UCLA-LS-3 [35].* This measure will also be used to characterise the level of self-reported parent/carer loneliness.

#### Process measures

*Goal based outcomes [47].* During their first intervention session young people will be asked to identify 3 intervention goals relating to their loneliness. They will be asked to rate on a 1–10 scale where they are in terms of achieving this goal; with 1 being “the furthest I could ever be from achieving this goal” and 10 “I have achieved this goal”. They will then rate each goal as part of the routine outcome measures for each session. Goal-based outcomes have been shown to improve treatment retention, clinical outcomes and client progress [62, 63].

*Visual analogue scales (VAS) [48].* For each session young people will be asked to rate their current mood, anxiety and loneliness on a 1–10 scale, where 10 is the worst. Visual analogue scales have been shown to have good validity and reliability [64].

#### Feasibility and experience measures

During the post-intervention assessment, the participants will be asked to complete the child and parent-report versions of the Experience of Services Questionnaire (ESQ) [39] regarding their experience of the intervention. Feasibility will be assessed based upon recruitment (meeting targets), retention, completion of measures and proportion of sessions attended by the participants. All participating families will also be asked how COVID-19 or other events have impacted the child’s loneliness during the intervention period. Finally, any adverse events that occur during the trial period will be recorded, reported and discussed within supervision.

### Data analysis

#### Primary outcome measure

The primary outcome measure of the SCED (self-reported scores on the Three Item Loneliness Scale [34]) will be analysed using visual inspection and statistical analysis.

For visual analysis, in accordance with SCED procedure, each participants data will be graphically represented on a line graph for the outcome variable [65, 66]. The baseline phase will be examined to determine a stable control. Data within and between each phase will then be compared for a) change in trend or symptom severity across phases, b) the degree of the slope on the graph which indicates the strength of the trend and c) change in the variability of the data to indicate stability in symptom change. Data will be mapped and inspected using both within-phase (evaluation of data patterns across participants in a single phase) and between-phase (comparison across adjacent phases for each participant) analyses [65, 66].

For statistical analysis Tau-U [45] will be used, which is a test specifically designed for single case research and has been used in previous SCEDs of psychological interventions [67, 68]. It is not dependent on distribution assumptions and calculations are based upon consideration of all data points. A further strength of the approach is that it can control for unwanted trends or variability in baseline scores to identify whether any change is the result of the introduction of the intervention [45]; making it a robust approach for SCED analysis. Tau-U will be used to analyse the overlap between the baseline and intervention phase and the baseline and post-intervention phase. The statistical analysis can be understood as the percentage of data that ‘improves’ across the phases of the study whilst considering any pre-existing baseline trends [68]. Tau-U will also be used to calculate an effect size and weighted average across all cases for the primary outcome variable.

#### Secondary outcome measures

It will be assessed how many of the participants display a reliable and clinically significant change [38] in total loneliness scores on the UCLA-LS-3 [35] and parent and self-reported impact scores on the SDQ [36]. It will also be examined how many participants display reliable change [38] in parent and self-reported Total Anxiety and Depression Scores on the RCADS [37], as well as how many participants report ‘clinically significant’ and ‘borderline clinical’ scores at baseline and post-intervention.

For the UCLA-LS-3 criterion B will be used to determine clinically significant change [38]. For criterion B, the participant must move within 2 standard deviations of the mean of a non-clinical comparison population. Criterion A and C will not be used due to lack of normative data available for a ‘clinically lonely’ population. The test-retest reliability used for the calculation will be 0.73 [35]. Normative data for the comparison population will be taken from a large-scale adolescent community sample, mean = 32.82, SD = 9.43 [49]. Reliable change will be calculated using Jacobson and Truax [38], whereby the baseline mean is subtracted from the post-intervention mean then divided by the standard error of measurement, giving the Reliable Change Index (RCI). The RCI indicates what would be categorised as clinically reliable improvement or deterioration.

For the RCADS well-defined norms are available [37], with raw scores being converted to age and gender-specific T-scores. A T-score of 65 means the young person is scoring in the top 7% for un-referred young people and is classified as “borderline clinical”. A T-score of 70 means that the young person is in the top 2% of unreferred young people and is described as the “clinical” threshold. The number of participants scoring within the clinical, borderline clinical and non-clinical ranges at baseline and post-intervention will be reported. As this current study has a small sample, the Reliable Change Index (RCI) used will be from a large normative sample [37].

For the SDQ parent and self-reported impact scores criterion C will be used to calculate clinically significant change. For criterion C, the participant’s score must move to the comparison side of the point halfway between the clinical and comparison group mean. Criterion C will be calculated as both clinical and comparisons norms are available. A test-retest reliability of .71 will be used [69]. For parent-report M = 0.4, SD = 1.1 will be used for comparison norms [36] and M = 6.2, SD = 2.7 for clinical norms [70]. For child report M = 0.2, SD = 2.8 will be used for comparison norms [36] and M = 5, SD = 3.3 [70] for clinical norms. For the SDQ impact scores, the Reliable Change Index (RCI) values will be based on those reported by Goodman [36] from a large normative sample.

The VAS and GBO’s will also be visually presented and the means and standard deviations of scores at baseline and post-intervention will be reported.

#### Feasibility and satisfaction measures

The proportion of our minimum recruitment target of 6 participants that we achieve will be reported. Successful completion of 6 participants will indicate that the recruitment protocol is feasible. The proportion of participants retained, defined as completing both the baseline and intervention assessments will be reported; with an 80% retention rate indicating that the research protocol is feasible, based on previous studies [71]. We will also report the proportion of appointments attended across all participants. The key themes identified in the Experience of Services Questionnaire [39] and the questionnaire assessing the impact of COVID-19 or other events on the child’s loneliness will also be reported. Acceptability will be indicated by 80% positive responses on the Experience of Services Questionnaire [39].

#### Data management, confidentiality and access

All data in the trial is anonymised. Participants will complete their questionnaires via Qualtrics [41], which is a password protected online platform. Consent forms will be stored on a separate encrypted database to participant data and linked by a unique participant ID number. Anonymised data will made available within the OSF data repository.

#### Patient public involvement (PPI)

A young person advisor and several parent/carer advisors provided advice on the study advertisements, information sheets, assessment protocol and intervention materials. A member of the University College London Loneliness and Social Isolation in Mental Health Research Network provided feedback on the intervention protocol. As the intervention protocol is being developed iteratively using PDSA cycles [31] it will also be adapted based upon within-session feedback from the participants.

## Discussion

To the best of our knowledge this will be the first study to test a CBT-based intervention for loneliness in young people ages 11–18. The intervention is modular and based upon Käll et al., [23] cognitive behavioural analysis of chronic loneliness. The intervention incorporates translated elements of Käll et al., [55] online intervention, and is informed by MATCH-ADTC [50], Groups4Health [51], PEERS social skills training [52] and CBT for Social Anxiety Disorder for adolescence [53]. The intervention will be evaluated through a SCED [28–30] of 6–8 participants. The primary objective of this study will be to test the efficacy of the intervention in terms of whether there are differences in scores on the Three-Item Loneliness Scale [34] between the different phases. The secondary objectives will be to examine whether there is reliable and clinically meaningful change [38] in self-reported loneliness scores on the UCLA-LS-3 [35], self and parent-reported impact scores on the SDQ [36] and self and parent-reported total scores on the RCADS [37]. The third objective will be to evaluate the feasibly and acceptability of the intervention. This will be defined in terms of a) meeting the recruitment target, b) participant retention and c) participant feedback of the intervention using the Experience of Service Questionnaire [39]. The results of the trial will be published in a peer reviewed journal and presented at relevant conferences.

### Limitations

The population of young people who are at an elevated risk of loneliness is highly heterogenous, [10, 11, 15, 16], and there is significant variability in the psychological problems across the 11–18 age range used in this study. The relatively small sample size means that it is not possible within this design to test the efficacy of the intervention broadly across these different subgroups. However, we suggest that the modular approach allows for the intervention to be applied flexibly, and so is feasible for use with the heterogenous population of young people presenting with chronic loneliness. If it is found that there is preliminary evidence that the modular intervention is effective, future research should examine the specific mechanisms maintaining loneliness for different subgroups of young people. This could allow for the development of bespoke adaptations of the intervention which may result in increased treatment engagement and efficacy.

The sample size used within this study is also relatively small and caution should be taken when interpreting the results. If preliminary evidence of effectiveness is found, an open trial followed by an RCT should be conducted to further examine the efficacy of the intervention, with RCT’s described as the ‘gold standard’ for effectiveness research [72].

Protocol version 1 (November 2021). Any changes to the protocol will be communicated to the journal and updated on ClinicalTrails.gov.

## Supporting information

S1 Checklist SPIRIT 2013 checklist: Recommended items to address in a clinical trial protocol and related documents*. (DOC)

S1 File (PDF)
