# Supplementary material for: The development of Cognitive Behavioural Therapy (CBT) for chronic loneliness in children and young people: Protocol for a single-case experimental design
Source: PLoS One. 2022 Dec 9;17(12):e0278746. doi: 10.1371/journal.pone.0278746 (PMC9733892; doi:10.1371/journal.pone.0278746)
Supplement: S1 File — (PDF) [file pone.0278746.s002.pdf]

Ethics Review Details

You have chosen to submit your project to the REC for review.	
Name:	Cawthorne, Thomas (2019)
Email:	NHJT027@live.rhul.ac.uk
Title of research project or grant:	The development of CBT for chronic loneliness in children
Project type:	Royal Holloway postgraduate research project/grant
Department:	Psychology
Academic supervisor:	Professor Andrew Macleod
Email address of Academic Supervisor:	A.Macleod@rhul.ac.uk
Funding Body Category:	No external funder
Funding Body:	
Start date:	01/02/2021
End date:	01/08/2022

Research question summary:

Chronic loneliness - lasting for three months or more - is defined as the discrepancy between actual and desired social interaction (Peplau & Perlman, 1982). It is linked with a variety of states, such as lower self-worth and greater emotional instability (Qualter, 2013). It is a transdiagnostic process associated with psychosis, suicidal behaviour, depression, and anxiety disorders (Mushtaq et al., 2014).). Approximately 10% of children and young people often experience loneliness and report that they 'often' feel lonely (Office for National Statistics, 2018). Interventions have been shown to reduce loneliness in young people as a secondary outcome (Eccles & Qualter, 2020). However, currently there are no interventions that specifically target chronic loneliness in young people. The meta-analysis concluded that whilst brief interventions promoting social skills and opportunities may be beneficial in reducing transient loneliness, interventions for chronic loneliness may require more complex interventions and the targeting of anxiety and cognitive biases which underlie chronic loneliness. A review of the literature has led to the development of a modular CBT model of loneliness and the identification of the effective practice elements of CBT interventions (Kall & Shafran, 2020). In the adult literature, CBT for loneliness has been shown to be effective, with a review of the different types of loneliness interventions by Masi et al (2011) identifying that the most efficacious interventions were those which targeted maladaptive social cognitions. However, currently such interventions for children and young people are lacking. Therefore, the purpose of this current study will be to develop and evaluate a CBT-based intervention which is designed specifically to target chronic loneliness in young people.

Research questions: Is a CBT-based intervention targeting chronic loneliness in children efficacious? Is it feasible to recruit and retain participants to the study? Do participants adhere to the intervention protocol and complete outcome measures?

Research method summary:

This study will be an online multiple-baseline single-case experimental design of CBT for chronic loneliness, in 6-8 young people ages 11-18 delivered over an average of 12 sessions. There will be three phases, A (baseline), B (intervention) and C (post-intervention). Participants will be recruited from schools, social media and word of mouth. Participants will attend a baseline eligibility assessment where they will complete a demographic questionnaire and parent and child-report measures of loneliness and mental health which should take an hour (see assessment measures document). To meet inclusion criteria young people will have to have been experiencing loneliness for more than 3-months and score above the mean on the UCLA Loneliness Scale (Russell, 1996). Participants will be excluded if there are significant risk issues, are currently receiving psychological therapy or are unable to access the intervention. Participants will receive a £25 voucher for completing the baseline assessment. Participants will be randomised to baseline lengths of 12-33 days. During the baseline phase participants will be asked to complete the 3 item +1 question UCLA loneliness scale and a diary of their social contacts daily, which should take 5 minutes per day. Participants will complete the same measures for each session throughout the intervention phase. During the post-intervention phase participants will be asked to complete the measures daily for a further 12 days. Participants will then complete a post-intervention assessment, where they will recomplete the questionnaire measures, given a feedback form on the intervention and asked how COVID-19/any other significant events have impacted their loneliness during the study. They will receive a £25 voucher for completing the post-intervention assessment. The primary outcome will be scores on the 3-item UCL loneliness scale. The results will be examined using visual analysis, Tau-U (Parker et al., 2011) and Jacobson's (Jacobson & Truax, 1992).

Risks to participants

Does your research involve any of the below?

Children (under the age of 16),

Yes

Participants with cognitive or physical impairment that may render them unable to give informed consent,

No

Participants who may be vulnerable for personal, emotional, psychological or other reasons,

Yes

Participants who may become vulnerable as a result of the conduct of the study (e.g. because it raises sensitive issues) or as a result of what is revealed in the study (e.g. criminal behaviour, or behaviour which is culturally or socially questionable),

No

Participants in unequal power relations (e.g. groups that you teach or work with, in which participants may feel coerced or unable to withdraw),

No

Participants who are likely to suffer negative consequences if identified (e.g. professional censure, exposure to stigma or abuse, damage to professional or social standing),

No

Details,

Participants will be young people (ages 11-18) and will include those with mental health conditions. The assessments and intervention will be conducted by Thomas Cawthorne, who is a Trainee Clinical Psychologist. He has a fully enhanced DBS check and several years experience working with children and young people. The study is supervised by Professors Roz Shafran and Andy Macleod who are both highly experienced Consultant Clinical Psychologists. During the intervention work, Thomas will received weekly supervision from Professor Roz Shafran.

During the consent process, we will get permission from all participants to inform their GP that they are involved in the study, as is good clinical practice. All participants will complete a baseline eligibility assessment. As part of this we will conduct a risk assessment and participants will not meet inclusion criteria if there are significant risk issues, e.g. significant suicidal ideation, previous suicide attempts, violence towards parents/siblings or significant safeguarding concerns with primary caregiver. If such issues were identified during the baseline assessment participants would be signposted to their GP and other appropriate agencies, e.g. CAMHS, and we would inform their GP of the risk issues identified by letter.

If risk issues are identified during the study, they would be discussed in the weekly supervision with Professor Roz Shafran and if necessary their GP will be informed and participants would sign posted to appropriate support agencies.

At the end of the study participants will be given a debrief sheet, where it will state that they should contact their GP if they require further support.

Design and Data

Does your study include any of the following?

Will it be necessary for participants to take part in the study without their knowledge and/or informed consent at the time?,

No

Is there a risk that participants may be or become identifiable?,

No

Is pain or discomfort likely to result from the study?,

No

Could the study induce psychological stress or anxiety, or cause harm or negative consequences beyond the risks encountered in normal life?,

No

Does this research require approval from the NHS?,

No

If so what is the NHS Approval number,

Are drugs, placebos or other substances to be administered to the study participants, or will the study involve invasive, intrusive or potentially harmful procedures of any kind?,

No

Will human tissue including blood, saliva, urine, faeces, sperm or eggs be collected or used in the project?,

No

Will the research involve the use of administrative or secure data that requires permission from the appropriate authorities before use?,

No

Will financial inducements (other than reasonable expenses and compensation for time) be offered to participants?,

No

Is there a risk that any of the material, data, or outcomes to be used in this study has been derived from ethically-unsound procedures?,

No

Details,

Risks to the Environment / Society

Will the conduct of the research pose risks to the environment, site, society, or artifacts?,

No

Will the research be undertaken on private or government property without permission?,

No

Will geological or sedimentological samples be removed without permission?,

No

Will cultural or archaeological artifacts be removed without permission?,

No

Details,

Risks to Researchers/Institution

Does your research present any of the following risks to researchers or to the institution?

Is there a possibility that the researcher could be placed in a vulnerable situation either emotionally or physically (e.g. by being alone with vulnerable, or potentially aggressive participants, by entering an unsafe environment, or by working in countries in which there is unrest)?,
No

Is the topic of the research sensitive or controversial such that the researcher could be ethically or legally compromised (e.g. as a result of disclosures made during the research)?,
No

Will the research involve the investigation or observation of illegal practices, or the participation in illegal practices?,
No

Could any aspects of the research mean that the University has failed in its duty to care for researchers, participants, or the environment / society?,
No

Is there any reputational risk concerning the source of your funding?,
No

Is there any other ethical issue that may arise during the conduct of this study that could bring the institution into disrepute?,
No

Details,

Declaration
By submitting this form, I declare that the questions above have been answered truthfully and to the best of my knowledge and belief, and that I take full responsibility for these responses. I undertake to observe ethical principles throughout the research project and to report any changes that affect the ethics of the project to the University Research Ethics Committee for review.

Certificate produced for user ID, NHJT027

Date:	08/02/2021 10:02
Signed by:	Cawthorne, Thomas (2019)
Digital Signature:	Thomas Cawthorne
Certificate dated:	08/02/2021
Files uploaded:	Assessment measures 03.02.2021.docx<br>Dclin child ascent form 31.01.2021.docx<br>DClin Debrief form 24.01.2021..docx<br>DClin Parent Consent from 03.02.2021.docx<br>DClin Thesis Parent Information Sheet 03.02.2021..docx<br>DClin Thesis Participant Information Sheet for YP 11-18 24.01.2021 version 2..docx<br>DClin Thesis Study Advert 01.02.2021- version 2.docx<br>References for ethics.docx
